# *Mycobacterium genavense*-induced spindle cell pseudotumor presenting with symptoms of giant cell arteritis

**DOI:** 10.1016/j.jdcr.2025.05.028

**Published:** 2025-06-17

**Authors:** Britney Le, Guilherme Kuceki, Jamie Zussman

**Affiliations:** a Ohio University Heritage College of Osteopathic Medicine, Dublin, OH; b Department of Dermatology, OhioHealth Riverside Methodist Hospital, Columbus, OH; c Department of Dermatology, University of Utah, Salt Lake City, UT

**Keywords:** acid-fast, giant cell arteritis, immunosuppression, mycobacteria

## Case description

An 80-year-old woman with poorly controlled diabetes (HbA1c 11.9%) presented with a painful, edematous, erythematous 1 cm nodule on the right temple. She reported a history of giant cell arteritis (GCA) 2 years earlier, for which she had received systemic corticosteroids but was lost to follow-up. A review of records revealed that a right temporal artery biopsy at that time was negative for arteritis. Due to ongoing concern for GCA, a temporal artery biopsy of the left side was obtained, which was also negative for arteritis. The right temple nodule was not biopsied at the time. Head computed tomography angiography showed a right temple subcutaneous soft tissue nodule and stranding, thought to be from the biopsy obtained 2 years earlier. Chest computed tomography revealed pulmonary calcified granulomas and calcified hilar lymphadenopathy, consistent with prior granulomatous disease. She was prescribed a systemic corticosteroid taper and referred to rheumatology, where a clinical diagnosis of GCA was made. Tocilizumab was initiated after latent tuberculosis, and Coccidioides were deemed unlikely. Notably, the interferon gamma release assay was twice indeterminate. Lung biopsy was deferred due to stability on repeat imaging.

**Fig 1:**
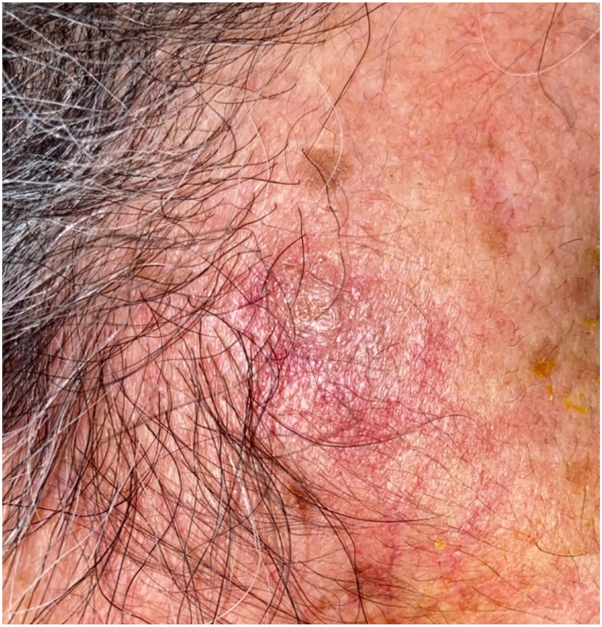
Clinical photo of the right temple at the time of first skin biopsy.

**Fig 2:**
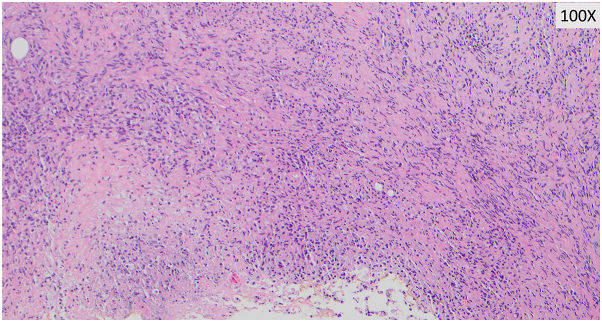
Dermal infiltrate of spindled histiocytes with interspersed lymphoplasmacytic inflammation.

**Fig 3:**
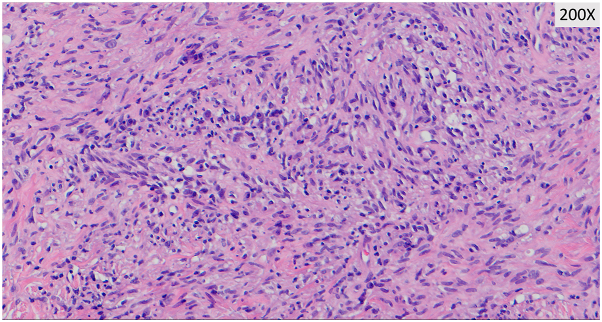
Higher magnifications reveal diffuse sheets of spindle-shaped histiocytes with foci of necrosis and leukocytoclasis.

**Fig 4:**
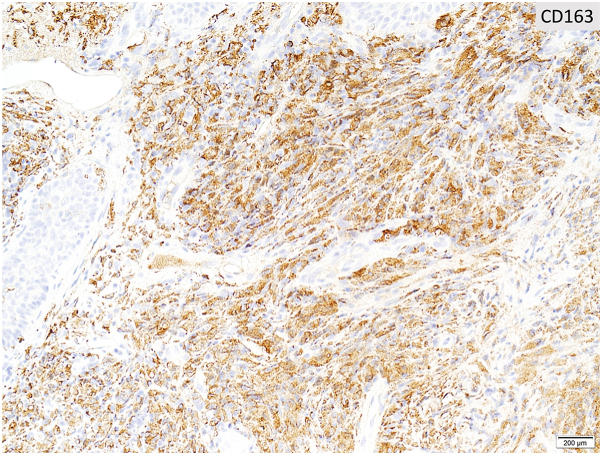
A CD163 is strongly positive in the histiocytes.

**Fig 5:**
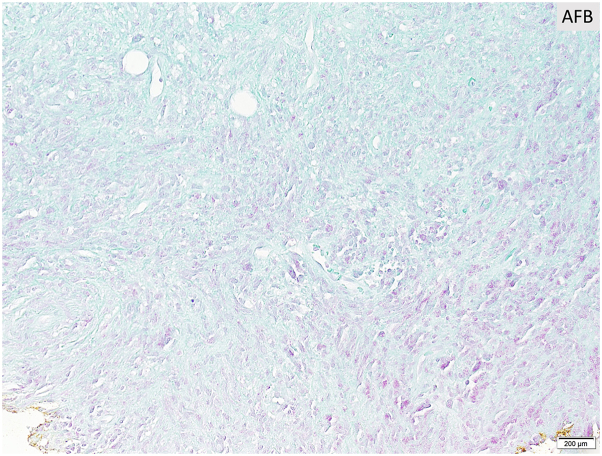
AFB stain highlights innumerable acid-fast bacilli at the base of the specimen within and surrounding the areas of necrosis. *AFB*, acid-fast bacillus stain.

After 3 months of tocilizumab and prednisone therapy, she returned with a right-sided headache and was treated for a presumed GCA flare. Two months later, during an admission for chest pain, she reported worsening pain and enlargement of the nodule on her temple. She denied recent dental, cosmetic, or surgical procedures prior to lesion development. Dermatology consult revealed a tender, red-brown papule overlying an indurated nodular plaque (Fig 1). Histopathology revealed diffuse spindled and epithelioid cells with necrosis, leukocytoclasis, and lymphoplasmacytic aggregates raising suspicion of mycobacterial infection (Figs 2 and 3). Immunohistochemistry was positive for CD163 (Fig 4), CD68, and CD31 and negative for AE1/AE3, SOX10, S100, CD34, and smooth muscle actin. Acid-fast bacillus stain showed abundant organisms (Fig 5); Gram and periodic acid–schiff were negative. Polymerase chain reaction confirmed *Mycobacterium genavense*. Tocilizumab was discontinued shortly after. She was treated with a combination antimicrobial therapy (rifabutin, moxifloxacin, azithromycin) for 12 months due to concern for disseminated infection. A follow-up biopsy demonstrated complete histologic resolution. She has now been off treatment for over 1 year, and her symptoms have remained completely resolved.

**Question 1: An 80-year-old woman with poorly controlled diabetes and a history of presumed GCA presents with a painful, enlarging right temple nodule. After multiple courses of corticosteroids and later tocilizumab, the lesion progresses. A biopsy months later reveals acid-fast bacilli and polymerase chain reaction confirms *Mycobacterium genavense*. Which of the following most likely contributed to the delayed diagnosis?**

**A.** Reliance on inflammatory markers such as erythrocyte sedimentation rate in guiding immunosuppressive therapy.
**B.** Misinterpretation of temporal artery biopsy results as falsely negative.
**C.** Diagnostic anchoring on prior presumptive GCA without investigating the evolving lesion.
**D.** Underappreciation of cutaneous mycobacterial infections in diabetic patients.
**E.** Failure to recognize imaging evidence of granulomatous disease as clinically relevant.

**Answer discussion:**

**C.** Correct. This case is unusual as the lesion’s location possibly contributed to symptoms mimicking GCA. It remains unclear whether her clinical course can be fully attributed to mycobacterial infection or if a component of GCA is also present; immunosuppressives likely contributed to the progression of the disease. Mycobacterial spindle cell pseudotumors are rare and most commonly caused by *Mycobacterium avium complex* or *Mycobacterium tuberculosis.*^1^ Their resemblance to neoplastic processes, such as Kaposi sarcoma or spindle cell melanomas, complicates diagnosis.^2^ *M. genavense* is a fastidious nontuberculous mycobacterium, often underdiagnosed due to its slow growth in culture. It is commonly found in gastrointestinal tracts and environmental sources, with infections primarily reported in patients with HIV or iatrogenic immunosuppression.^3^ Early recognition and accurate diagnosis of mycobacterial spindle cell pseudotumor are crucial, as *M. genavense* infections carry a mortality rate of ∼30% but are curable in over 50% of cases with timely treatment.^4^^,^^5^

## Conflicts of interest

None disclosed.
